# Adherence and Attrition in Public Fluoride Varnish Programs: A Mixed‐Methods Systematic Review

**DOI:** 10.1111/ipd.70144

**Published:** 2026-09-15

**Authors:** Nathan Zeyang‐Liu, Rahul Gupta, Joseph Chiang, Jolanta Aleksejuniene

**Affiliations:** ^1^ Faculty of Dentistry University of British Columbia Vancouver British Columbia Canada

## Abstract

**Background:**

Public fluoride varnish (FV) programs, often delivered as part of a multi‐component oral preventive package, rely on the patient's continued participation. However, continued adherence is often poor in real‐world settings.

**Aim:**

To synthesize child, family, provider, and systemic level barriers and facilitators influencing adherence and attrition in public FV programs for children < 18 years in multi‐ethnic high‐income countries.

**Design:**

Systematic review was conducted in accordance with PRISMA 2020. Six databases were searched for studies reporting barriers and facilitators to repeated FV applications.

**Results:**

From 3616 records, 22 studies were included. Child‐level barriers included behavioral distress, sensory aversion, and temporary medical exclusions. Family‐level barriers included caregiver capacity, health literacy, and mobility. Provider‐level barriers included workforce constraints, training, role boundaries limiting task‐sharing, and weak school/community coordination. System‐level barriers included inequitable reach to high‐risk groups and geographic constraints. Facilitators included culturally safe, relationship‐based engagement, task‐sharing, school/community‐based delivery with active follow‐up systems, and child‐centered engagement strategies. Meta‐analysis demonstrated a pooled attrition rate of 23% (95% CI 8%–43%, *n* = 14 455).

**Conclusion:**

Adherence and attrition in public FV programs are shaped by interacting determinants across child, family, provider, and system levels. Retention should be considered when designing and implementing these programs.

**Trial Registration:**

CRD420251044425 (PROSPERO)

## Why This Paper Is Important to Pediatric Dentists

- Identifies that approximately one in four children who receive an initial fluoride varnish application do not return for the second application.
- Describes how barriers across child‐, family‐, provider‐, and systemic‐level domains may interact to influence adherence and attrition in recurring preventive oral health interventions.
- Highlights practical strategies and facilitators that support sustained participation in publicly delivered oral health programs.

## 1 Introduction

Dental caries remains one of the most common chronic conditions in children and a persistent public‐health challenge. In 2017, an estimated 532 million children worldwide had untreated caries in primary teeth and 2.3 billion people had caries in permanent teeth [1]. Despite being largely preventable, dental caries disproportionately affects Indigenous and minority children in multi‐ethnic high‐income countries (USA, Canada, UK, Australia), who consistently experience earlier disease onset, greater severity, and higher rates of untreated caries compared with their non‐minority peers [2, 3]. These inequities reflect longstanding social, cultural, and structural determinants of health, including socioeconomic marginalization, colonial histories, reduced access to preventive services, and systemic barriers to receiving routine dental care [4]. Addressing these disparities is a key priority in the current landscape of dental public health.

Fluoride varnish (FV) has long occupied a prominent role in professional fluoride therapy for dental caries prevention in children. Clinical guidelines have recommended its application at regular intervals for children starting at the age of primary teeth eruption [5, 6, 7]. The evidence underpinning these recommendations, however, has narrowed in recent decades. More recent synthesis indicates that FV's anticaries effect in preschoolers is more modest and less certain than previously assumed, with limited additional benefit for children already using fluoride toothpaste [8, 9]. Economic evaluations have raised similar concerns regarding FV, with no convincing evidence that FV application in preschoolers is cost‐effective in settings where children are already exposed to fluoridated water or toothpaste [10]. Nevertheless, to expand reach and reduce access barriers, many health systems have integrated public FV delivery as a part of a multi‐component oral preventative package that includes supervised toothbrushing, sealants, atraumatic restorative treatments (ART), and oral health education into community, school‐based, and primary‐care settings [11]. Such programs are typically directed at higher caries risk populations with limited access to private dental care, where school‐based delivery has been shown to increase utilization and reduce unmet dental treatment needs [12]. All of these components, like FV, require families to sustain contact with the program over time. Low adherence to recommended schedules, attrition, and the high cost of running public oral health programs therefore constrain how these programs deliver care and limit their capacity to reduce oral health disparities.

Although general barriers to dental service use are well described, the specific factors that influence continued participation in public FV programs in multi‐ethnic high‐income countries remain insufficiently understood. Existing evidence is scattered across heterogeneous study designs, and no systematic review has synthesized how child, family, provider, and systemic level factors interact as barriers and facilitators to repeated FV application. A clearer understanding of these factors is needed to strengthen downstream preventive programs and to ensure that they are well supported by the broader upstream conditions necessary for sustained participation.

This mixed‐methods systematic review aims to identify and synthesize the child, family, provider, and systemic level barriers and facilitators influencing adherence and attrition in public FV programs for children under 18 years in multi‐ethnic high‐income countries. By integrating qualitative and quantitative evidence through a mixed‐methods approach, this review outlines how multilevel domains may interact to shape sustained participation and attrition for repeated FV applications.

## 2 Materials and Methods

### 2.1 Protocol

This review was registered in the International Prospective Register of Systematic Reviews (PROSPERO) and was conducted in accordance with the Joanna Briggs Institute methodology for mixed‐methods systematic reviews and the Preferred Reporting Items for Systematic Reviews and Meta‐Analyses (PRISMA) 2020 guidelines [13, 14].

### 2.2 Deviation From Protocol

Several deviations from the registered PROSPERO protocol occurred as the review progressed. First, the population and setting criteria were broadened. The protocol restricted inclusion to children aged 0–6 years in public fluoride varnish programs in the United States and Canada. During initial screening, it became clear that this scope excluded a substantial body of relevant evidence, particularly studies involving school‐aged children and public programs implemented in comparable multi‐ethnic high‐income countries. To appropriately address the review question, the criteria were expanded to include children under 18 years in publicly delivered FV programs and to include studies from the United States, Canada, the United Kingdom, and Australia.

Second, the protocol anticipated a purely qualitative narrative synthesis. However, the final evidence base included several studies reporting comparable quantitative outcomes (e.g., attrition and participation rates). In response, an inverse‐variance weighing random‐effects meta‐analysis was conducted. Conversely, qualitative data were analyzed using an inductive thematic synthesis rather than a narrative summary, to allow more rigorous handling of heterogeneous study designs.

Third, the final search window was set to January 2015 to July 2025 to ensure contemporary relevance, given substantial changes in public fluoride varnish delivery strategies over the last decade, as well as evolving patterns in child behavior, family dynamics, and engagement with preventive health services that are likely to influence adherence and attrition in real‐world settings.

### 2.3 Eligibility Criteria

Eligibility criteria included studies involving children less than 18 years of age, participating in publicly delivered fluoride varnish programs and studies delivered through community or school‐based settings in high‐income, multi‐ethnic countries (USA, Canada, UK, and Australia). Studies were eligible if they reported author or participant‐identified barriers, social determinants, and factors influencing adherence and attrition in continued FV program participation. Letters to the editor, commentaries, review articles, editorials, and conference abstracts were excluded from our review. Non‐English publications were also excluded.

### 2.4 Search Strategy and Information Sources

A systematic search of six databases, Medline, Embase, Web of Science, CINAHL, Scopus, and CENTRAL, was completed on July 21 and 22, 2025. Studies published from January 1, 2015, to July 21, 2025, were included. An initial search strategy was developed by study authors N.Z.‐L. and R.G. and subsequently verified in collaboration with a medical librarian from the University of British Columbia. Controlled vocabulary (e.g., MESH, Emtree) and free‐text terms were used and adapted for each database. Search concepts included fluoride varnish and topical fluoride, dental caries and early carious lesions, and pediatric populations. Full search strategies for all databases are provided in the appendix.

### 2.5 Study Selection

All search results were imported into Covidence (Veritas Health Innovation Ltd.), which automatically removed duplicate studies. Two reviewers (N.Z.‐L. and R.G.) independently screened abstracts and titles against the inclusion criteria, with conflicts resolved through discussion. Studies deemed relevant proceeded to full‐text review, which was conducted using the same independent review process. Reviewers were not blinded to journal titles, study authors, or research institutions.

### 2.6 Data Extraction

Data extraction from included studies was organized using Google Sheets (Google LLC). A structured data extraction form was developed by N.Z.‐L. prior to data collection based on predetermined variables and outcomes of interest. For each eligible study, the following information was charted: reference, study design, country, publication year, study setting, population description, sample size, consent procedure, FV frequency, other combined interventions, delivery mode, provider type, attrition rate, non‐participation rate, author and participant reported child, family, provider, and system‐level barriers and facilitators.

### 2.7 Risk of Bias Assessment

Study quality was independently evaluated by two reviewers (N.Z.‐L. and R.G.) with disagreements resolved by discussion. Quality was assessed using version 2 of the Cochrane Risk of Bias tool for randomized controlled trials (RoB 2) and the ROBINS‐I tool for observational studies (Figures 1, 2) [15, 16].

**FIGURE 1:**
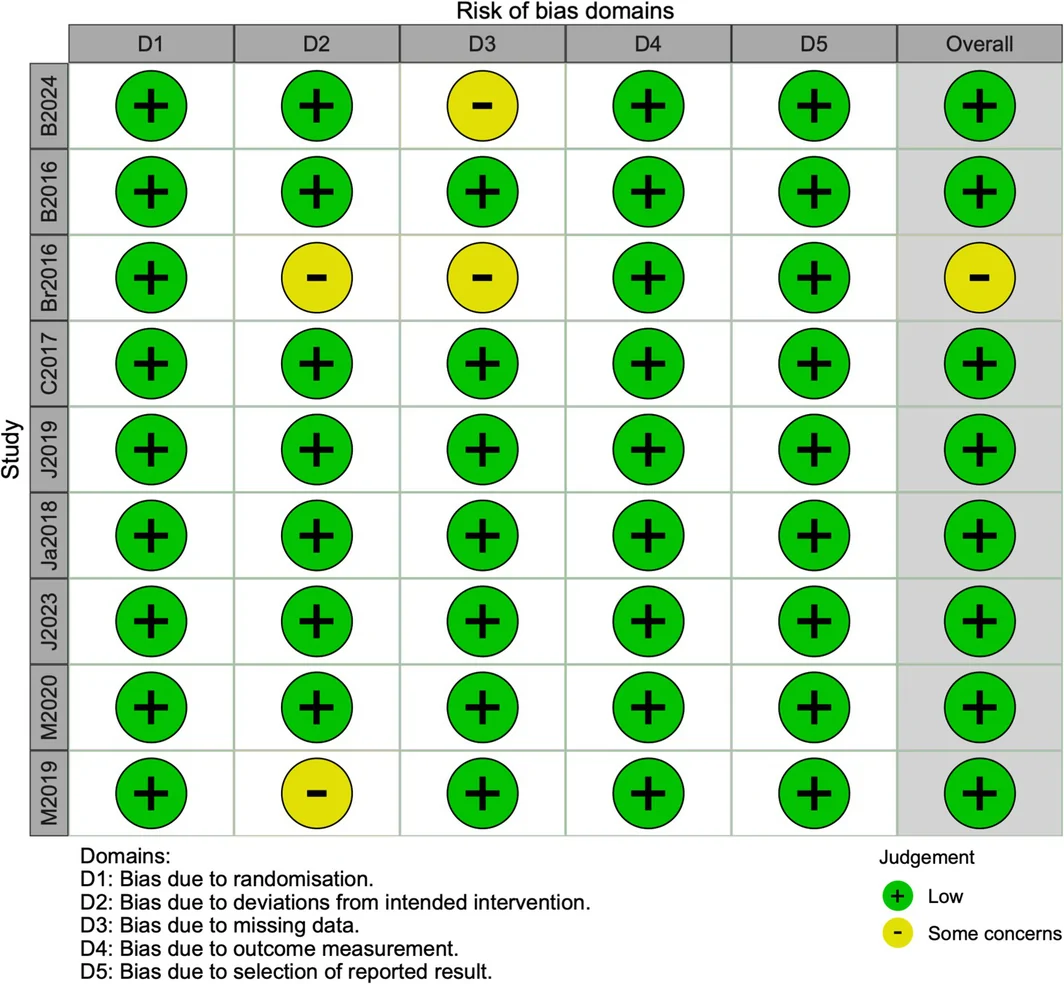
Risk of bias assessment of randomized controlled trials. The traffic‐light plot displays domain‐level judgments for each study across five domains: Bias arising from the randomization process (D1), bias due to deviations from intended interventions (D2), bias due to missing outcome data (D3), bias in measurement of the outcome (D4), and bias in selection of the reported results (D5).

**FIGURE 2:**
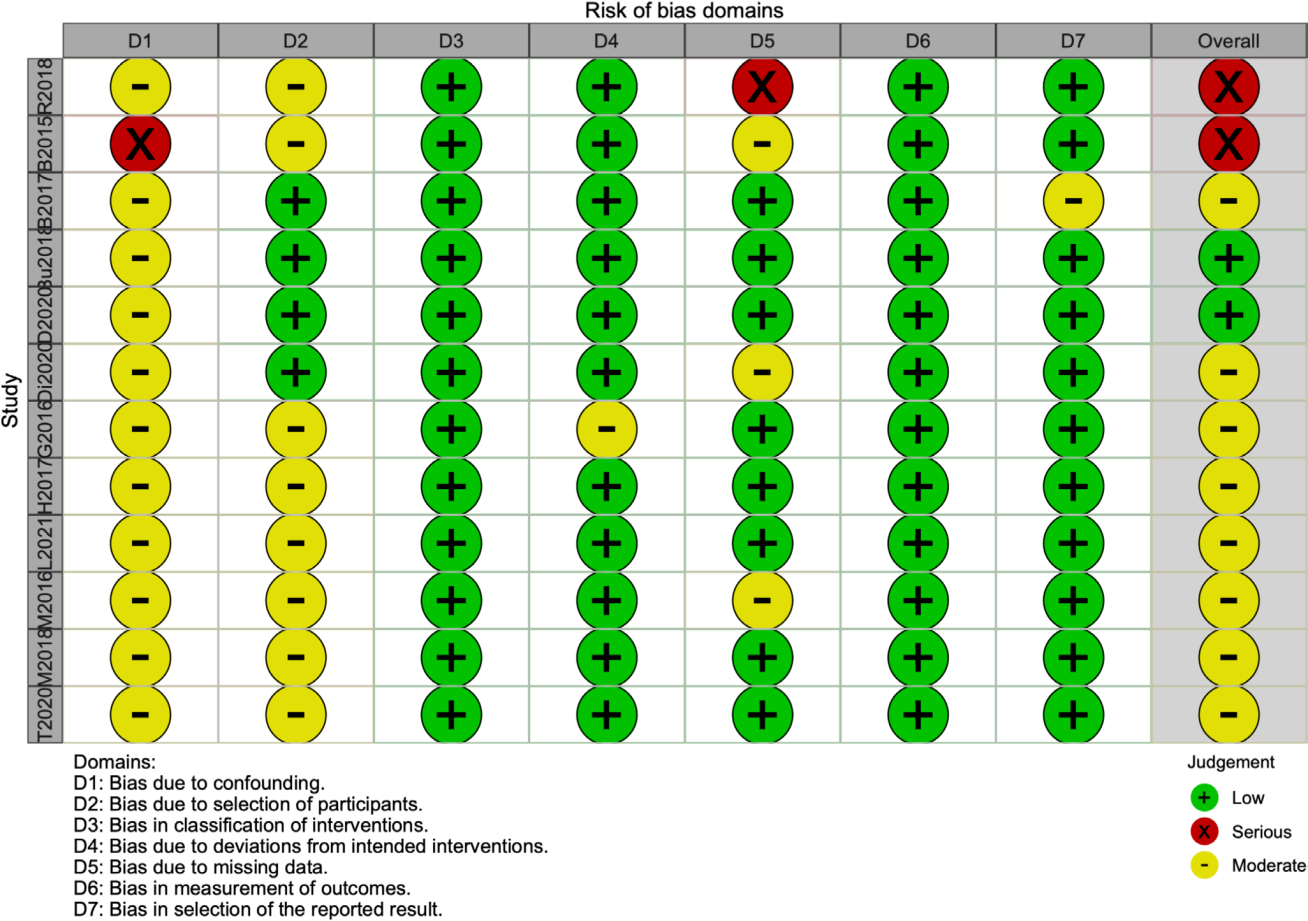
Risk of bias in non‐randomized studies of interventions. Traffic‐light plot presenting domain‐level judgments across seven domains: bias due to confounding (D1), bias in selection of participants (D2), bias in classification of intervention (D3), bias due to deviations from intended intervention (D4), bias due to missing data (D5), bias in measurement of outcomes (D6), and bias in selection of reported result (D7).

### 2.8 Knowledge Synthesis

A mixed‐methods approach was used to synthesize qualitative and quantitative findings. Qualitative findings were synthesized thematically to identify multilevel barriers and facilitators influencing adherence and attrition in public FV programs. Quantitative data were pooled to estimate attrition rate between sequential FV applications.

### 2.9 Qualitative Synthesis

Qualitative data were synthesized using an inductive thematic analysis following the approach described by Braun and Clarke [17, 18]. Qualitative data consisted of secondary author‐reported descriptions of barriers and facilitators extracted from the results and discussion sections of included studies.

Relevant text segments describing factors influencing continued participation, adherence, or attrition in each study's results and discussion sections were extracted verbatim. Extracted data were initially categorized into four predefined domains: child‐level, family‐level, provider‐level, and system‐level barriers and facilitators.

Subsequently, inductive coding was conducted to identify patterns across domains. Coding and theme development were led by N.Z.‐L. using NVivo version 15 (QSR International). An iterative, line‐by‐line coding process was employed, involving familiarization with the data, generation of initial codes, grouping of codes into candidate themes, and refinement and naming of final themes. Themes were reviewed to ensure internal coherence and clear conceptual distinction. Discrepancies in interpretation were discussed with co‐authors to enhance analytic rigor.

### 2.10 Quantitative Synthesis

Quantitative synthesis focused on estimating attrition rate between sequential FV applications. Attrition was defined as the proportion of children who successfully received the first FV application but did not attend the second FV application. Studies were eligible for quantitative pooling if they reported sufficient data to calculate the attrition rate.

Nine studies met the criteria for inclusion in the quantitative synthesis. A meta‐analysis of proportions was conducted using inverse‐variance weighting to estimate the pooled attrition proportions across studies. Proportions were stabilized using the Freeman–Tukey double arcsine transformation. A random‐effects model was applied to account for heterogeneity between studies. Study variance was estimated using the Hartung‐Knapp method. Results were summarized graphically using forest plots displaying individual study attrition proportions and corresponding 95% confidence intervals. Due to the small number of included studies, subgroup and publication bias analyses were not feasible.

## 3 Results

### 3.1 Study Selection

A total of 3616 records were identified through database searching. After removal of 2045 duplicates, 1571 unique records remained for title and abstract screening. Of these, 1442 records were excluded based on titles and abstracts, leaving 129 articles for full‐text assessment. Following full‐text review, 107 studies were excluded for the following reasons: studies not involving publicly delivered fluoride varnish programs (*n* = 4), studies conducted in non‐eligible countries (*n* = 13), studies reporting irrelevant outcomes (*n* = 26), inappropriate study design (*n* = 25), or full texts not available (*n* = 39). A total of 22 studies met the inclusion criteria and were included in the final review. The study selection process is summarized in Figure 3.

**FIGURE 3:**
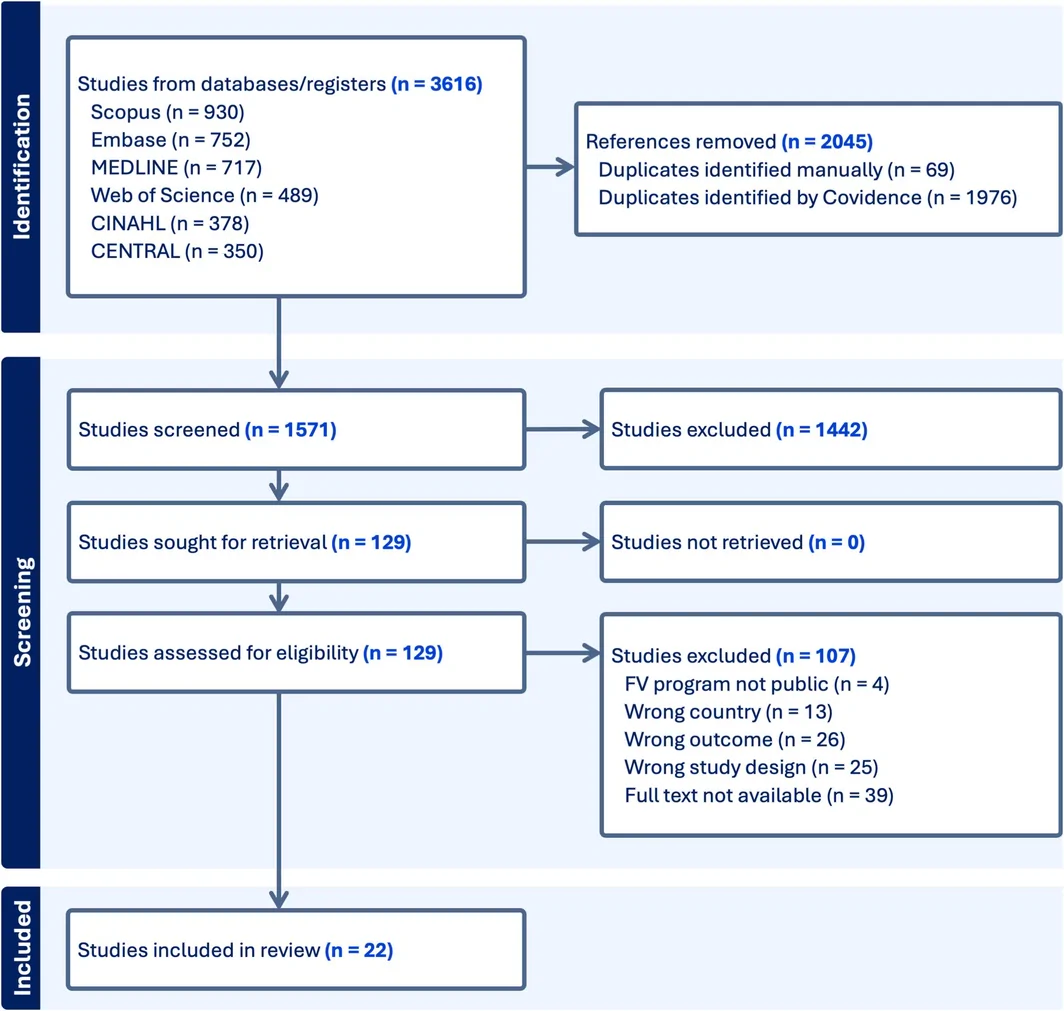
PRISMA 2020 flowchart.

A summary of study characteristics can be found in Table 1. The median year of publication was 2019 (interquartile range [IQR] 3; range 2015–2025). Studies were from Australia (*n* = 7), USA (*n* = 6), UK (*n* = 5), and Canada (*n* = 4). Most interventions were carried out in school‐based and community health settings. The majority of studies focused on socially disadvantaged populations, including low socioeconomic status families, Indigenous populations, and children from minority or rural communities. Participant age ranged from infancy to 17 years of age. A summary of study interventions and outcome of interest can be found in Table 2.

**TABLE 1:** Summary of study characteristics of included articles.

Study ID	Study title	Study design	Country	Setting	Population	Sample size
Ruff and Niederman (2018)	Comparative effectiveness of school‐based caries prevention: a prospective cohort study.	Prospective cohort study	USA	Public title I elementary schools	Low‐SES primary school children aged 5–12 years	8207
Barry Godin et al. (2024)	Screening and treatment time in school‐based caries prevention: A randomized clinical trial.	Longitudinal, pragmatic, cluster‐randomized, non‐inferiority trial	USA	Title I equivalent public primary schools	Low‐SES and minority (black and/or hispanic/latino) primary school children (average age of 7.6)	7418
Biordi et al. (2015)	Improving access and provision of preventive oral health care for very young, poor, and low‐income children through a new interdisciplinary partnership.	Longitudinal demonstration project	USA	One urban and one rural Women, Infants, and Children (WIC) program sites	Children younger than 5 years whose parents or guardians were enrolled in the WIC program (average age: 2.31, 3.15, 3.76 at the first, third and fifth visit, respectively)	4360
Braun et al. (2016)	A cluster‐randomized, community‐based, tribally delivered oral health promotion trial in Navajo Head Start Children.	3 year longitudinal cluster‐randomized community‐based trial	USA	Navajo Head Start centers (52 in total; 26 intervention and 26 usual care)	Navajo children aged 3–5 years in the Head Start program	897
Bryant et al. (2016)	A community‐based oral health intervention in Navajo Nation Head Start: Participation factors and contextual challenges.	Cluster‐randomized community‐based clinical trial	USA	Navajo Head Start centers (52 in total; 26 intervention and 26 usual care)	Navajo children (average age of 3.6) in the Head Start program. Rural, low‐income communities	518
Buckingham and John (2017)	Outcomes and costs of preschool and school‐based fluoride varnish pilots.	Uncontrolled pilot study/feasibility evaluation	UK	Preschool and primary school settings in areas of deprivation	School children age 3–7 years old	589
Burgess‐Allen et al. (2018)	Challenges associated with implementation of a school‐based toothbrushing and fluoride varnish program in a diverse and transient urban population.	Pilot/feasibility evaluation	UK	Six culturally diverse, disadvantaged schools in Derby City	Children aged 3–5 years in nursery and reception classes	455
Chestnutt et al. (2017)	Fissure seal or fluoride varnish? A randomized trial of relative effectiveness.	Phase IV, two parallel‐arm, randomized clinical trial	UK	66 primary schools in “Communities First” areas (social and economic deprived areas)	Children aged 6–7 years with at least one fully erupted first permanent molar	1016
Dimitropoulos et al. (2020)	A school fluoride varnish program for Aboriginal children in rural New South Wales, Australia.	Small scale feasibility study of a school‐based fluoride varnish (FV) program	Australia	3 rural schools with majority Aboriginal children	Aboriginal children aged 5–17 years old	104
Dimitropoulos et al. (2020)	Outcomes of a co‐designed, community‐led oral health promotion program for Aboriginal children in rural and remote communities in New South Wales, Australia.	Consecutive surveys comparing oral health status and hygiene behaviors in 2018 against baseline data collected in 2014	Australia	Three schools (two “Moderately Accessible”, one “Remote”) in Central Northern New South Wales (NSW)	Aboriginal children aged 5–12 years old	88
Ghazal et al. (2016)	Dental caries in high‐risk, school‐age African American Children in Alabama: A six‐year prospective.	Secondary data analysis of a 6‐year prospective cohort study	USA	One primary school in the “Black Belt” county of Perry, Alabama	6‐year‐old African American Children	98
Huber et al. (2017)	Redirecting public oral health fluoride varnish intervention to low socioeconomic status children in Alberta.	Descriptive analysis of the Alberta Health Service (AHS) Oral Action Plan	Canada	Community health centers, libraries, daycares, parent centers and schools within the AHS jurisdiction	Low‐SES children divided into two groups: Preschool aged 12–35 months Kindergarten, Grade 1, and Grade 2	Preschool: 5710 School‐age: 25813
Jamieson et al. (2019)	Follow‐up of an intervention to reduce dental caries in indigenous Australian children: A secondary analysis of a randomized clinical trial.	Secondary analysis of a 2‐arm parallel, outcome assessor‐masked, randomized clinical trial	Australia	Recruitment occurred through hospital antenatal clinics and Aboriginal Community Controlled Health Organizations	Pregnant women who identified as being pregnant with an Aboriginal and/or Torres Strait Islander child; follow‐up included their children up to 3 years of age	448
Jamieson et al. (2018)	Dental disease outcomes following a 2‐year oral health promotion program for Australian aboriginal children and their families: A 2‐arm parallel, single‐blind, randomized controlled trial.	Single‐blind, parallel‐arm, randomized controlled trial	Australia	Recruitment conducted through health service providers	Women pregnant with an Aboriginal child residing in South Australia	448
Ju et al. (2024)	Early childhood caries intervention in aboriginal Australian children: A cross‐in randomized trial.	Outcome assessor‐blinded, closed‐cohort cross‐in randomized controlled trial	Australia	Recruitment within hospitals	Women pregnant with an Aboriginal child residing in South Australia	448
Lalloo et al. (2021)	Carious lesions in permanent dentitions are reduced in remote Indigenous Australian children taking part in a non‐randomized preventive trial.	Non‐randomized trial	Australia	A remote Indigenous community in Far North Queensland	Indigenous school‐going children aged 5–17 years	408
Mathu‐Muju et al. (2016)	The Children's Oral Health Initiative: An intervention to address the challenges of dental caries in early childhood in Canada's First Nation and Inuit communities.	Program assessment and description of a community‐based public‐health intervention	Canada	Federal reserves and remote communities in the Yukon, Northwest Territories, and Nunavut	Preschool children from birth to 4 years of age, school children (5–7 years of age)	23 585 (in 2012)
Mathu‐Muju et al. (2018)	Utilization of community health workers in Canada's Children's Oral Health Initiative for indigenous communities	Secondary data analysis prospectively collected over a 7‐year longitudinal study period	Canada	Isolated Northern Indigenous reserves	Children in three age cohorts: 0–23 months, 2–4 years, and 5–7 years of age	21 174 (over a 7 year period)
McMahon et al. (2020)	Fluoride varnish in nursery schools: A randomised controlled trial—protecting teeth.	Double‐blind, two‐arm randomized controlled trial	UK	Nursery schools located within the areas of four NHS Health Boards (Greater Glasgow and Clyde, Fife, Lothian, and Tayside)	Children aged 3 years in their first nursery year	1916
Morgan‐Trimmer et al. (2019)	The acceptability of fluoride varnish and fissure sealant treatments in children aged 6–9 delivered in a school setting.	Two‐arm randomized controlled trial	UK	Primary schools in designated Community First areas with high levels of socioeconomic disadvantage	Children aged 6–9 years	1016
Tadakamadla et al. (2020)	Surface‐specific caries preventive effect of an intervention comprising fissure sealant, povidone‐iodine and fluoride varnish in a remote indigenous community in Australia.	Non‐randomized clinical trial	Australia	Remote Indigenous community in the Northern Peninsula Area of Far North Queensland	School children aged 4–17 years	408
Yerex et al. (2025)	Children's Oral Health Initiative program's impact on First Nations and Inuit children.	Retrospective analysis of a national population‐based oral health prevention program database from 2006 to 2016	Canada	Federal reserves and remote Indigenous communities in Atlantic Canada (New Brunswick, Newfoundland and Labrador, Nova Scotia, and Prince Edward Island), Saskatchewan, and Ontario	Registered First Nations and Inuit children (aged 0 to 7 years)	80 574

**TABLE 2:** Summary of study intervention and outcomes.

Study ID	Consent procedure	FV application frequency	Other interventions	Delivery mode	Provider type	Attrition	Non‐participation at baseline
Ruff and Niederman (2018)	Paper parent/guardian consent returned to school	Twice yearly (6‐monthly)	Sealants, prophy, oral hygiene education	School‐based program	Hygienists (prevention), dentists (examinations)	NR	70% non‐participation at baseline
Barry Godin et al. (2024)	Parental informed consent and child assent	One allocation appointment and one follow‐up	Experimental Arm: Fluoride Varnish to all teeth Control Arm: Atraumatic restorations on all frank, asymptomatic, cavitated lesions and fluoride varnish to all teeth	School‐based program with mobile equipment setup	Dental hygienists or registered nurses under supervision by dentist	0% attrition rate	~59% non‐participation at baseline
Biordi et al. (2015)	Parental/Guardian informed consent and custom designed fourth‐grade reading level consent forms for participants	Visits spaced 3 or 6 months apart up to 5 visits	Oral health assessment, parent/guardian education, referral to pediatric dentists, provision of toothbrush, toothpaste, age‐appropriate education materials, dietary counseling	Clinic‐based program at WIC sites	Registered dietitian, nursing and dietetics students trained by a pediatric nurse practitioner	58% attrition rate	NR
Braun et al. (2016)	Written consent and assent	Four fluoride applications during each school year	Oral health promotion (OHP) events for children and caregivers. Toothbrushes and toothpaste distributed.	School‐based program in Head Start Classrooms	Community oral health specialists (8 trained tribal community members)	NR (81.2% of children in the intervention group received 3 or more fluoride varnish applications)	17% non‐participation at baseline for intervention group 16% non‐participation at baseline for usual care group
Bryant et al. (2016)	Not reported explicitly	Four fluoride varnish applications during each school year	Oral health promotion (OHP) events for children and caregivers.	School‐based program in Head Start Classrooms	Community oral health specialists (trained native lay workers)	Cohort 1 (2011): had an attrition rate of 4.7% Cohort 2 (2012): had an attrition rate of 5.5%	3.3% non‐participation at baseline
Buckingham et al. (2017)	Consent and complete medical history forms	Three fluoride varnish applications per school year	Daily supervised toothbrushing sessions Oral health education for children, parents, and school staff Dental screening to assess decay levels and needs Signposting/referral letters for children needing treatment	School‐based program in preschools and primary schools	Extended‐duty dental nurse application with dentist supervision	7.2% attrition rate	22.2% non‐participation at baseline
Burgess‐Allen et al. (2018)	Parental consent with assistance of translated material and pictorial aids	Two fluoride varnish application within an 18‐month pilot period	Supervised daily toothbrushing in school Take‐home brushing kits Educational curriculum packs for children, parents, staff Baseline and follow‐up dental examinations	School‐based (in nursery and reception classrooms)	Dental team member	56% attrition rate	2% non‐participation at baseline
Chestnutt et al. (2017)	Written consent from parent or guardian	6 month intervals for 36 months	Comparator arm received pit and fissure sealants	School‐based delivery with mobile dental clinics	Qualified and trained dental hygienists	Fluoride varnish arm: 4% attrition rate	22% non‐participation at baseline
Dimitropoulos et al. (2020)	Written consent from parents or guardian with verbal consent from child prior to every application	3 month intervals for 12 months	Co‐designed activities: daily in‐school toothbrushing regular dental health education delivery of oral hygiene resources encouraging water consumption over sugary drinks	School‐based program with portable dental chairs	Oral health therapists	NR	54% non‐participation at baseline
Dimitropoulos et al. (2020)	Written consent was obtained from parents or guardians with verbal assent from each child before screenings or questionnaires	3 month intervals	Daily supervised in‐school toothbrushing Water bottle program with refrigerated/filtered fountains Regular distribution of toothpaste and brushes for home use Oral health education on hygiene and diet Any dental treatment as required	School‐based	Oral health therapists	NR	15% non‐participation at baseline
Ghazal et al. (2016)	Informed consent was obtained from the children's caregivers	6 month intervals for 2 years, then 12 month intervals thereafter	Oral hygiene instructions Anticipatory guidance, Preventive oral health kits (toothbrush, fluoride toothpaste, floss, mirror) Referrals to dentists for observed cavitated lesions	School‐based with portable equipment	Dentist, dental hygienists and dental residents	NR	NR
Huber et al. (2017)	Preschool: direct consent from parents/legal guardian School‐age: parents received consent form which must be signed and returned	Preschool: 2 applications per year over a 2‐year period School‐age: 2 applications per year	Fluoride varnish only but part of the Oral Action Health Plan which includes sealants and oral health training for care providers of older adults	Preschool: Community‐based (health centers, libraries, daycare, parent centers) School‐age: School‐based during school hours	Registered dental assistants and hygienists	Preschool: 47% attrition rate School‐age: 16% attrition rate	Preschool: NR School‐age: 45% non‐participation at baseline
Jamieson et al. (2019)	Written informed consent	Immediate intervention (II): FV at child ages 6, 12, and 18 months. Delayed intervention (DI): FV at child ages 24, 30, and 36 months	Dental treatment for mothers during pregnancy Motivational interviewing Anticipatory guidance on oral health education	Community‐based/home visits	Trained study staff	II: 9% attrition rate DI: 0% attrition rate	33% non‐participation at baseline (not explicitly reported)
Jamieson et al. (2018)	NR	Three applications at child age 6, 12, and 18 months old	Dental treatment for mothers during pregnancy Motivational interviewing Anticipatory guidance on oral health education	Community‐based//home visits	Trained study staff	9% attrition rate	33% non‐participation at baseline (not explicitly reported)
Ju et al. (2024)	Written informed consent	Three applications for each group: at 6, 12, and 18 months for the immediate group; and at 24, 30, and 36 months for the delayed group	Dental treatment for mothers during pregnancy Motivational interviewing Anticipatory guidance on oral health education	Community‐based/home visits	NR	Immediate group: 9% attrition rate Delayed group: 0% attrition rate	33% non‐participation at baseline
Lalloo et al. (2021)	Written informed consent for the study with additional written consent obtained according to Queensland Health standard procedures if treatment was necessary	Annual FV application	Fissure sealants Application of povidone‐iodine Completion of necessary restorative dental treatment Provision of standard diet and oral hygiene advice	School‐based with mobile unit; hospital dental clinics	Dentists, oral health therapists, and dental assistants	35% attrition rate	32% non‐participation at baseline
Mathu‐Muju et al. (2016)	Collective consent at the community level; informed consent from parents of children receiving intervention	Initial fluoride varnish application with subsequent preventative care services throughout programmatic year	Oral health screening Fissure sealants Oral health counseling/education Atraumatic restorative therapy (ART) with glass ionomers for active decay	School‐based clinics and community‐based home visits	Dental therapists, dental hygienists, and Children's Oral Health Initiative aides	NR	NR
Mathu‐Muju et al. (2018)	Community wide consultation and consent; informed consent from parents of children receiving intervention	Initial fluoride varnish application with re‐application every 3–4 months	Oral health screening Fissure sealants Oral health counseling/education Atraumatic restorative therapy (ART) with glass ionomers for active decay	Community‐based home visits, events, and clinical appointments	Dental professionals (dental therapists, hygienists, and dentists) and Children's Oral health Initiative aides	NR	NR
McMahon et al. (2020)	Parental/guardian informed consent	6 month intervals (baseline, 6, 12, and 18 months) over a 24 month period	All children received Treatment‐As‐Usual (TAU) Childsmile components (e.g., daily supervised toothbrushing, home packs, community support, dental‐practice prevention)	School‐based within nurseries	Treatment teams	15% attrition rate	33% non‐participation at baseline
Morgan‐Trimmer et al. (2019)	Parental consent forms with child assent verbally before interviews	6 month intervals over a 36 month period	Fissure sealants Oral hygiene education via the National Oral Health Improvement Programme “Designed to Smile”	School‐based via mobile dental clinics	Community Dental Service (dental hygienists and nurses)	NR	NR
Tadakamadla et al. (2020)	Two‐stage informed consent: initial consent for interview‐based questionnaire and epidemiological examination and subsequent consent for interventions	Annual application for two consecutive years	Fissure sealants Povidone‐iodine 10% Restoration of cavitated lesions Toothbrush/toothpaste provision	School‐based examinations on portable dental chairs with interventions conducted in local hospital clinics or a mobile dental van	Trained oral health professionals (dentists and oral health therapists)	NR	NR
Yerex et al. (2025)	Permission to analyze granted by the Department of Indigenous Services Canada and regional First Nations and Inuit Health Branch offices	Program guidance of a minimum of two applications per year recommended for high‐risk children	Annual oral health screenings Dental sealants Atraumatic restorative treatment Interim stabilization treatment Oral health education/counseling	Community‐based delivery	Dental therapists, dental hygienists, Children's Oral Health Initiative aides and non‐oral health providers with limited training	NR	NR

### 3.2 Qualitative Analysis

Thematic synthesis identified multilevel barriers and facilitators influencing adherence and attrition in public fluoride varnish (FV) programs and studies. Barriers existed at child, family, provider, and systemic levels, often interacting to shape participation across repeated FV applications. Identified themes and their descriptions are summarized in Table 3.

**TABLE 3:** Thematic synthesis of child, family, provider, and systemic level barriers and facilitators influencing adherence and attrition in publicly delivered fluoride varnish programs.

Domains	Themes	Description	Contributing studies
Child‐level barriers	Theme 1.1: Emotional and behavioral barriers	Dental anxiety, behavioral distress, negative anticipatory experiences, and lack of verbal assent on the day of application reduced children's cooperation and acceptance of fluoride varnish, limiting successful application and willingness to participate in subsequent visits.	Morgan‐Trimmer 2019 [19], Dimitropoulos 2020 [20], McMahon 2020 [21], Godin 2024 [22]
	Theme 1.2: Sensory experience barriers	Aversion to the taste, texture, or oral sensation of fluoride varnish, including gag reflex and heightened sensitivity limited children's tolerance of the procedure and affected repeat participation.	Morgan‐Trimmer 2019 [19], Tadakamadla 2020 [23], Yerex 2025 [24]
	Theme 1.3: Temporary or medical exclusions	Acute illness or rare medical contraindications temporarily prevented fluoride varnish application, resulting in missed or delayed treatments that occasionally disrupted participation.	Buckingham 2017 [25], Huber 2017 [26], McMahon 2020 [21]
Family‐level barriers	Theme 2.1: Caregiver capacity and competing life demands	High household demands, caregiver stress, and competing social or economic priorities constrained families' capacity to consistently engage with preventive oral health programs over time.	Braun 2016 [27], Bryant 2016 [28], Buckingham 2017 [25], Burgess‐Allen 2018 [29]
	Theme 2.2: Family engagement and health literacy barriers	Limited caregiver understanding of fluoride varnish benefits, low oral health literacy, and complex or burdensome consent procedures reduced program uptake, follow‐up participation, and sustained engagement.	Biordi 2015 [30], Braun 2016 [27], Buckingham 2017 [25], Burgess‐Allen 2018 [29], Jamieson 2018 [31], Ruff 2018 [32], Dimitropoulos 2020 [33], Tadakamadla 2020 [23], Lalloo 2021 [34], Yerex 2025 [24]
	Theme 2.3: Structural and economic barriers to access	Transportation challenges, economic hardship, school absenteeism, and transitions related to aging out of eligibility limited families' ability to sustain participation in fluoride varnish programs.	Biordi 2015 [30], Braun 2016 [27], Bryant 2016 [28], Burgess‐Allen 2018 [29], Jamieson 2018 [31], Dimitropoulos 2020 [20], Lalloo 2021 [34]
	Theme 2.4: Cultural safety, trust, and social identity	Stigma associated with targeted programming, mistrust of health systems, and cultural discordance between families and providers reduced program acceptability and engagement, particularly among Indigenous and minority populations.	Braun 2016 [27], Huber 2017 [26], Mathu‐Muju 2018 [35]
	Theme 2.5: Family mobility and discontinuity of care	Frequent family relocation or changes in school enrollment disrupted continuity of care and contributed to loss to follow up.	Chestnutt 2017 [36], Chestnutt 2017 [37], Huber 2017 [26], Burgess‐Allen 2018 [29], Jamieson 2018 [31], Mathu‐Muju 2018 [35], Ruff 2018 [32], Dimitropoulos 2020 [33], Dimitropoulos 2020 [20], McMahon 2020 [21], Lalloo 2021 [34], Godin 2024 [22]
Provider‐level barriers	Theme 3.1: Workforce capacity and resource constraints	Shortages of dental professionals, high administrative workload, and limited staffing resources restricted program reach and delivery consistency of fluoride varnish services.	Biordi 2015 [30], Mathu‐Muju 2016 [4], Buckingham 2017 [25], Huber 2017 [26], Burgess‐Allen 2018 [29], Mathu‐Muju 2018 [35], Dimitropoulos 2020 [20], McMahon 2020 [21], Lalloo 2021 [34], Godin 2024 [22], Yerex 2025 [24]
	Theme 3.2: Training, skill, and confidence requirements	Variability in provider training, skill, and confidence, particularly in treating young children, contributed to inconsistent application practices and affected follow‐up participation.	Biordi 2015 [30], Ruff 2018 [32], Godin 2024 [22]
	Theme 3.3: Role clarity and professional boundaries	Scope‐of‐practice restrictions limited provider autonomy and task‐sharing, confining independent fluoride varnish application to only dentists and dental hygienists in some settings despite the feasibility of delivery by trained non‐dental healthcare providers.	Mathu‐Muju 2016 [4], Morgan‐Trimmer 2019 [19], Dimitropoulos 2020 [33], Godin 2024 [22]
	Theme 3.4: Relationship and communication challenges with schools and communities	Weak or inconsistent collaboration with school personnel and community leaders, particularly in settings without established trust, hindered program incorporation, coordination, and continuity of fluoride varnish delivery.	Buckingham 2017 [25], Chestnutt 2017 [37], Huber 2017 [26]
	Theme 3.5: Program structure and scheduling limitations	Poorly designed program structures and limited delivery days limited access to care and reduced opportunities for repeated fluoride varnish applications.	Burgess‐Allen 2018 [29], Mathu‐Muju 2016 [4], Mathu‐Muju 2018 [35], Tadakamadla 2020 [23], Yerex 2025 [24]
System‐level barriers	Theme 4.1: Inequitable reach and coverage	Children at highest risk for dental caries, including preschool‐aged children, those in rural communities, and marginalized populations, were under‐reached by public fluoride varnish programs.	Buckingham 2017 [25], Huber 2017 [26], McMahon 2020 [21]
	Theme 4.2: Geographic and access constraints	Ruralness, remoteness, and transportation barriers limited access to preventive oral health services.	Biordi 2015 [30], Bryant 2016 [28], Mathu‐Muju 2016 [4], Mathu‐Muju 2018 [35], Lalloo 2021 [34]
	Theme 4.3: Weak system coordination and continuity pathways	Fragmented service delivery, poor communication, and inadequate follow‐up infrastructure contributed to discontinuity of care across fluoride varnish delivery programs.	Bryant 2016 [28], Huber 2017 [26], Ruff 2018 [32]
	Theme 4.4: Program scheduling challenges	In school delivered fluoride varnish delivery programs, disruptions to school schedules, instructional time, and graduations interfered with continuous fluoride varnish delivery.	Morgan‐Trimmer 2019 [19], Godin 2024 [22]
Facilitators of repeated FV application	Theme 5.1: Culturally sensitive and relationship‐based engagement	Culturally safe, trust‐based engagement with families and communities, including collaboration with school staff, community leaders, and community health workers, enhanced program acceptability, sustainability, and follow‐up, particularly in Indigenous and minority populations.	Braun 2016 [27], Bryant 2016 [28], Mathu‐Muju 2016 [4], Buckingham 2017 [25], Huber 2017 [26], Burgess‐Allen 2018 [29], Jamieson 2018 [31], Mathu‐Muju 2018 [35], Jamieson 2019 [38], Morgan‐Trimmer 2019 [19], Dimitropoulos 2020 [33], Dimitropoulos 2020 [20], Ju 2024 [39], Godin 2024 [22], Yerex 2025 [24]
	Theme 5.2: Expanded workforce capacity through shared delivery roles	Task‐sharing with trained non‐dental providers, such as school nurses and non‐dental community healthcare workers, supported by standardized training protocols, increased workforce capacity and enabled more consistent delivery of repeated fluoride varnish applications.	Biordi 2015 [30], Bryant 2016 [28], Mathu‐Muju 2018 [35], Jamieson 2019 [38], Morgan‐Trimmer 2019 [19], Tadakamadla 2020 [23], Lalloo 2021 [34], Godin 2024 [22], Yerex 2025 [24]
	Theme 5.3: Access‐enhancing service delivery and follow‐up systems	School and community‐based delivery models, transportation supports, integration with existing public‐health infrastructure, and active follow‐up and communication systems improved access, attendance, and continuity of care.	Biordi 2015 [30], Braun 2016 [27], Bryant 2016 [28], Mathu‐Muju 2016 [4], Buckingham 2017 [25], Chestnutt 2017 [37], Chestnutt 2017 [36], Huber 2017 [26], Burgess‐Allen 2018 [29], Ruff 2018 [32], Jamieson 2019 [38], Morgan‐Trimmer 2019 [19], Dimitropoulos 2020 [20], Lalloo 2021 [34]
	Theme 5.4: Child‐centered approaches to comfort and engagement	Positive reinforcement, child‐appropriate positioning techniques, oral health education, and motivational interviewing strategies improved children's comfort, cooperation, and willingness to participate in repeated fluoride varnish applications.	Biordi 2015 [30], Buckingham 2017 [25], Morgan‐Trimmer 2019 [19], Dimitropoulos 2020 [20], Ju 2024 [39]

#### 3.2.1 Child‐Level Barriers

Child‐level barriers were primarily related to children's emotional responses and sensory experiences associated with fluoride varnish application. Across studies, anxiety, distress, and behavioral resistance, particularly among younger children, were commonly reported as limiting successful application and willingness to participate in subsequent visits. Sensory aversion to the taste or texture of fluoride varnish further compounded these challenges, particularly for children with heightened oral sensitivity or gag reflexes. Temporary exclusions due to acute illness or medical considerations also contributed to missed applications, occasionally disrupting participation.

#### 3.2.2 Family‐Level Barriers

Family‐level barriers included caregiver capacity, competing life demands, and socioeconomic stressors which constrained families' ability to prioritize repeated preventive visits, even when initial participation occurred. Limited caregiver understanding of fluoride varnish benefits and complex consent processes further reduced follow‐up participation. Structural challenges, including transportation barriers, school absenteeism, and aging out of the program, contributed to loss to follow up, while family mobility and changes in school enrollment disrupted continuity of care. Issues related to cultural safety and trust in health systems were particularly noticeable in studies involving Indigenous and marginalized communities.

#### 3.2.3 Provider‐Level Barriers

Provider‐level barriers reflected constraints related to workforce capacity, training, and program organization. Staffing shortages, high administrative workload, and limited dedicated time for preventive services restricted the consistency and reach of fluoride varnish delivery. Variability in provider training, skill, and confidence, particularly in managing young children, contributed to inconsistent applications and influenced follow‐up participation. In several settings, regulatory scope‐of‐practice restrictions and unclear professional boundaries limited provider autonomy and task‐sharing, confining fluoride varnish application to dentists or dental hygienists despite its suitability for delivery by trained non‐dental healthcare providers. In addition, weak or inconsistent relationships between providers, schools, and communities, particularly in settings without established trust, constrained program continuity and reduced the reach and effectiveness of fluoride varnish delivery.

#### 3.2.4 System‐Level Barriers

System‐level barriers were characterized by inequitable program reach, access limitations, and fragmented service delivery. Several studies reported that children at highest risk for caries were disproportionately underrepresented in fluoride varnish programs, particularly preschool‐aged children and those in marginalized or remote communities. Geographic remoteness, transportation challenges, and limited infrastructure further constrained access. In school‐based programs, disruptions related to academic schedules, transitions between grades or schools, and limited follow‐up contributed to incomplete fluoride applications.

#### 3.2.5 Facilitators of Repeated Fluoride Varnish Applications

Facilitators of sustained participation emphasized relationship‐based, flexible, and access‐enhancing approaches. Culturally sensitive engagement strategies that fostered trust with families and community leaders supported program acceptability and retention, particularly in Indigenous settings. Expanding workforce capacity through task‐sharing with trained non‐dental providers improved delivery capacity. School and community‐based models, integrated follow‐up systems, and proactive communication with caregivers enhanced attendance and continuity. Child‐centered strategies, including positive reinforcement and comfort‐focused techniques, further supported repeated participation.

### 3.3 Meta‐Analysis

Nine studies provided sufficient data to quantify attrition and were included in the meta‐analysis. Attrition was defined as the proportion of children who received an initial fluoride varnish application but did not attend the subsequent scheduled application.

Across included studies, attrition estimates varied widely, ranging from 3% to 58%. Using an inverse‐variance model, the pooled attrition rate between the first and second applications was estimated at 23% (95% CI 8%–43%, *n* = 14 455) (Figure 4). Substantial between‐study heterogeneity was observed (*I* ^2^ = 99.7%), reflecting differences in study populations, program settings, delivery models, and follow‐up structures.

**FIGURE 4:**
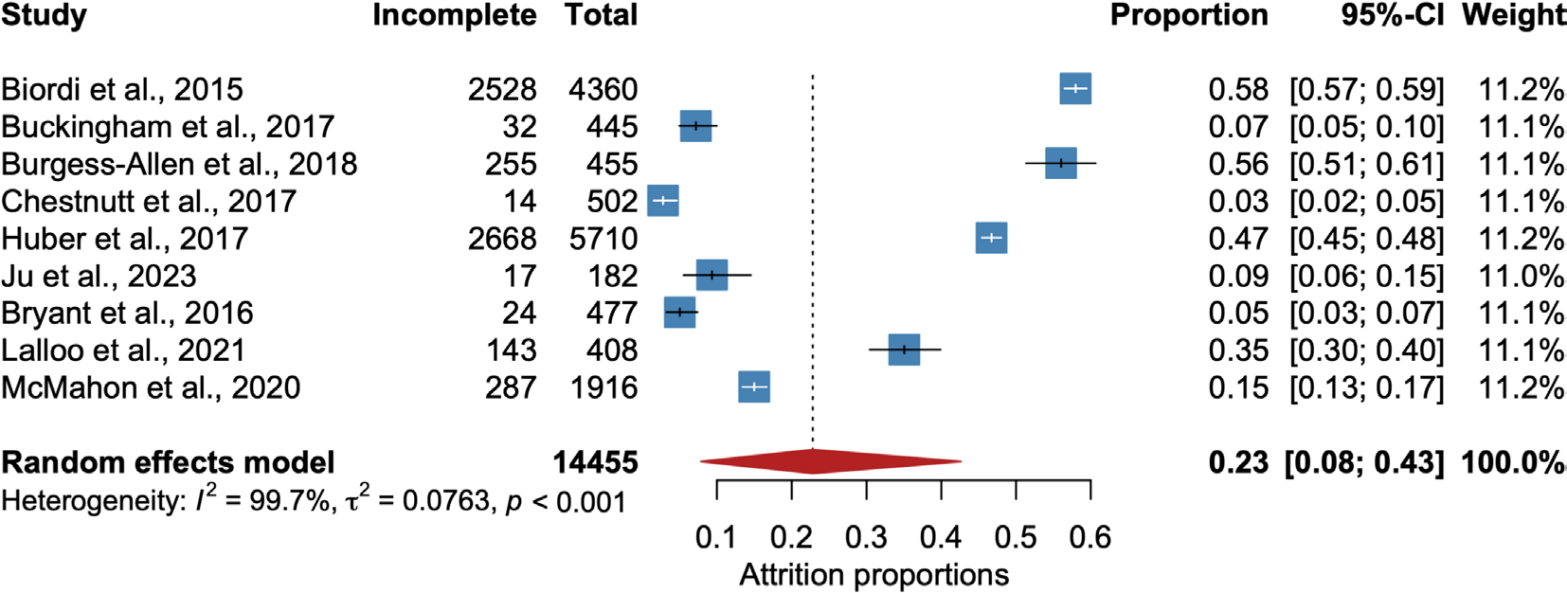
Attrition (proportions) across public fluoride varnish programs. Forest plot of individual study attrition (incomplete participation following first fluoride varnish application) and corresponding 95% confidence intervals. Proportions were pooled using a random‐effects model with Freeman–Tukey double arcsine transformation. Diamond represents the pooled attrition proportion and width indicates 95% confidence intervals.

Given the small number of studies and the high degree of heterogeneity, subgroup analyses were not conducted. The pooled estimate should therefore be interpreted as an overall summary of observed attrition rather than a precise population‐level rate.

## 4 Discussion

This mixed‐methods systematic review found that approximately one‐in‐four children who receive an initial FV application did not attend the subsequent scheduled application. The accompanying thematic synthesis indicates that the loss to follow up is attributable to interacting variables across the child, family, provider, and systemic level domains. Some of these barriers included the children's emotional and sensory responses at the point of application, caregiver capacity and health literacy, family mobility, workforce and scope‐of‐practice constraints, and systemic level limitations in program reach and follow‐up infrastructure. Attrition in public FV programs is therefore socially and structurally produced, rather than solely attributable to the caregiver or child.

The significance of this finding should be considered alongside the evolving evidence base for FV effectiveness. In 2013, the American Dental Association (ADA) recommended applying 2.26% FV at least every three to six months for children at elevated risk of developing caries [5]. The ADA restricted its recommendations to higher‐risk populations, noting that lower risk patients may not need topical fluorides beyond over the counter fluoridated water and toothpaste. The panel cautioned, however, that some of its evidence predated the 1970s, when caries rates were higher and fewer people received fluoridated water or toothpaste. It therefore advised caution in extrapolating these findings to today's high‐risk populations and called for research on the effectiveness of topical fluorides at the current levels of background fluoride exposure.

Recent evidence has begun to address this gap, though it remains contested. The 2013 Cochrane review reported a substantial caries preventive effect of FV, a 37% reduction in decayed, missing, and filled surfaces in the primary dentition [7]. Subsequent reviews incorporating newer and lower risk of bias trials have reached a more cautious conclusion. de Sousa et al. [9], incorporating trials published after 2013, concluded that FV had a modest and uncertain anticaries effect in preschoolers, with only about 12% protection on average and was considerably inconsistent across trials. They observed that FV was less effective in more recently published trials than in older ones, a pattern they attributed to the higher risk of bias in older studies that overestimated treatment effects. An overview of reviews by Couto et al. (2026) reached a similar conclusion, finding that most existing systematic reviews of FV effectiveness are of critically low quality and at a higher risk of bias [8]. The reviews incorporating more recent trials showed that FV added little to no preventive benefits for children who are already using fluoride toothpaste. Finally, a systematic review of economic evaluations found no convincing evidence that FV application in preschoolers is cost‐effective in settings where children are already exposed to fluoridated water or toothpaste [10].

Taken together, this evidence may support positioning FV as one component of a comprehensive preventive care package rather than as the intervention around which public oral health programs are organized. In practice, this is how many oral health programs are already structured. Table 2 shows that nearly every program included in this review delivered FV within a multi‐component package alongside supervised toothbrushing, sealants, atraumatic restorative treatment, and oral health education (20 of 22 studies).

The barriers identified in our synthesis are not specific only to FV. Because they interact across multiple domains to influence whether families sustain contact with preventive services, they would be expected to shape participation in any repeated oral health intervention delivered to these populations. This pattern is especially evident in higher‐risk northern Canadian Indigenous communities, where geographic and structural barriers to reliable, repeated access to dental care mean that the children at highest caries risk are the least consistently reached by routine preventive services [40]. The wide range of attrition observed across included studies (3%–58%) likely reflects program heterogeneity, consistent with evidence that retention in preventive health programs is strongly context dependent, shaped by local delivery conditions and access to care [41]. Understanding how these multilevel determinants affect retention is therefore necessary to improve both the clinical effectiveness and the cost‐effectiveness of public oral health programs.

Our thematic synthesis reveals that attrition in public FV programs is driven by a chain of linked processes. From acceptance by children, through caregiver engagement and provider execution, to continuity within the delivery system, each step can break down and lead to loss to follow up. This is consistent with emerging evidence showing that barriers of FV delivery span multiple domains [42].

At the first encounter, the children's emotional state and early behavioral responses influence procedural cooperation during the fluoride varnish application. Zhou et al. (2013) demonstrated that pre‐procedure anxiety predicted increased future disruptive behaviors (Theme 1.1, 1.2), while early cooperative behaviors predicted continued engagement. The authors recommend encouragement‐centered guidance, reframing dental instruments as engagement tools, and allowing children to hold and explore instruments to reduce perceived threat and enable their curiosity‐driven cooperation. These findings highlight the value of positive behavior reinforcement to sustain child engagement, with tell‐show‐do often referenced as a supportive approach [43]. FV programs are strengthened when clinical encounters are intentionally shaped around the children's comfort, agency, and their developmental stage (Theme 5.4).

Extending beyond the interaction level, Christian et al. [44] emphasized that the public‐health impact of FV delivery increases when preventive interventions are embedded within familiar, trusted, and non‐threatening community primary‐care platforms and supported by a trained, expanded non‐dental workforce. Their review identifies caregiver trust and procedural acceptability as key facilitators of preventive service uptake (Theme 3.4). Together, these findings reinforce that child‐centered care should be a core principle in FV program implementation in order to support early cooperation and sustained participation.

Beyond the child, caregiver capacity and competing family demands (Theme 2.1) emerged as major barriers. Implementation research in preventative oral health highlights that household complexity, competing responsibilities, and practical constraints, including time, transportation, and workflow disruption (Theme 2.3), shape caregivers' ability to sustain follow‐up participation in repeated interventions [42]. One strategy identified to alleviate household burden was delivering FV in settings where children already spend time, particularly schools and daycare centers, which were consistently recognized as a key structural facilitator. (Theme 5.3). By leveraging existing infrastructure, these co‐located delivery models may reduce logistical burdens, including transportation barriers and caregiver scheduling constraints, thereby improving program reach and increasing opportunities for completing repeated FV applications. Supporting this, Griffin et al. [45] identified schools as an important access‐enhancing platform for FV delivery, particularly for children from socially deprived or low‐income households, with school‐based FV programs generating approximately threefold higher annual FV exposure compared to schools without such programs.

Limited oral health literacy and misconceptions about fluoride varnish risks contribute to incomplete perceptions of FV's preventive value (Theme 2.2). Evidence shows that one‐in‐two caregivers demonstrate partial, inaccurate, or absent understanding of fluoride varnish benefits, reinforcing the need for simple and literacy‐sensitive communication strategies to support caregiver preventive decision‐making and sustained patient engagement [46].

Integration of fluoride delivery within existing primary‐care and public‐health services strengthens follow‐up consistently by embedding FV use as a part of regular healthcare encounters (Theme 5.2, 5.3). Christian et al. [44] argue that preventative oral health programs achieve meaningful public‐health impact when delivery systems are integrated into primary care and designed for continuity, proactive patient recall, workforce sharing, and child‐centered acceptability, because retention and repeated dosing are socially and structurally determined, not individually driven.

Several programs addressed program‐level limitations by expanding the pool of personnel able to deliver FV (Theme 5.2). Task‐sharing models leverage non‐dental professionals, such as physicians, nurses, community health workers, or trained volunteers to apply varnish. This can greatly increase program capacity and extend geographic reach. For example, an interdisciplinary program in Ohio, USA used nurse practitioner and dietitian teams to conduct oral health assessments and deliver thousands of repeated FV applications across multiple visits, improving preventive care access for young children in low‐income families [30]. This aligns with evidence from Canada's Children's Oral Health Initiative (COHI), where local community health workers supported home outreach, caregiver communication, scheduling, and repeated FV delivery, particularly through home visits, school‐based programs, and community follow‐ups. Importantly, COHI implementation data showed that continuity of community health worker availability produced consistently higher enrollment and delivery of multiple FV applications, sealants, and ART, while sporadic worker availability was associated with persistently low enrollment and low repeated fluoride delivery over time [35]. Community healthcare workers often hold strong community trust and are able to deliver oral health counseling and culturally concordant preventive education, enabling caregiver understanding and their participation [47]. Training and standardized clinical protocols are foundational to effective task‐sharing in preventive oral health programs, ensuring that FV can be applied safely, consistently, and be scaled up by community‐based or health personnel [35]. The task‐sharing model strengthens overall delivery capacity and reduces the workload put on any single provider group.

Finally, a recurring facilitator (Theme 5.1) was the use of culturally sensitive engagement and strong relationship building with communities. Programs that invested in trust‐building, for example, by involving Indigenous elders, community leaders, or bilingual staff aides, reported better acceptance and follow‐up among families [48]. In Indigenous communities, Indigenous‐delivered or community‐led oral health initiatives helped address longstanding mistrust of government health services by respecting local customs and empowering local personnel (Theme 2.4). For instance, Canada's Children's Oral Health Initiative (COHI) deploys community‐based dental therapists and aides, who share the cultural background of the families, to provide preventive care in Indigenous communities. COHI staff reported that culturally appropriate parental engagement and education were crucial for continuous support, and that when parents understood the program in the context of their community values, they were more likely to keep their children enrolled [47]. These findings reflect the broader evidence‐based recommendations that improving providers' cultural competence and involving community members in program design can increase the trust of Indigenous and minority populations and reduce access barriers [49]. In practice, this might mean offering interpretation services, tailoring health messages to resonate with cultural beliefs, and respecting cultural norms during program delivery. Trust, once established, tends to reinforce itself. Families who have positive, respectful encounters with the program staff are more likely to return and encourage their peers to participate.

The limitations of this systematic review are as follows. First, the qualitative synthesis relied on secondary data, specifically, researchers reported participant barriers presented in the results and discussion sections of individual studies, rather than on direct primary data from participants themselves. This approach may introduce interpretive and reporting biases from the original study teams and may not fully capture the participants' perspectives.

Second, only 9 of the 22 included studies contributed data to the meta‐analysis. The pooled attrition estimate should therefore be interpreted as an exploratory rather than a generalizable population rate. Additionally, participation and attrition metrics were reported inconsistently across studies, differing in fluoride varnish application intervals, outcome definitions, and reporting formats. This variability highlights the need for more standardized data reporting. Future research would benefit from standardized reporting guidelines focused on adherence and attrition outcomes.

In conclusion, public FV programs depend on the families' sustained contact over repeated applications. This review found that roughly one‐in‐four children do not return for a second application. Adherence and attrition are shaped by interacting factors across child, family, provider, and systemic domains, and are socially and structurally produced rather than attributable solely to the caregiver or child. Because these determinants are not specific to FV, they may extend to any repeated oral health preventive intervention delivered to these populations. Retention should be considered as a core outcome in the design and implementation of oral health prevention programs serving high‐risk children.

## Author Contributions

N.Z.‐L., R.G., and J.A. conceptualized the research design and methodology. R.G. conducted the literature review. N.Z.‐L., R.G., and J.C. carried out data collection. N.Z.‐L. and R.G. conducted data analysis, interpreted findings, and drafted the manuscript. J.A. provided methodological guidance, oversight throughout the study, and revised the final manuscript. All authors read and approved the manuscript and agree to be accountable for all aspects of the work.

## Funding

The authors have nothing to report.

## Disclosure

The authors have nothing to report.

## Conflicts of Interest

The authors declare no conflicts of interest.

## Supporting information


**Appendix S1:** Full search strategies for all databases are provided in the appendix.

**Data S1:** PRISMA 2020 Checklist.

## Data Availability

The data that support the findings of this study are available from the corresponding author upon reasonable request.
