# Supplementary material for: Adherence and Attrition in Public Fluoride Varnish Programs: A Mixed‐Methods Systematic Review
Source: Int J Paediatr Dent. 2026 Sep 15;36(6):758–82. doi: 10.1111/ipd.70144 (PMC13712569; doi:10.1111/ipd.70144)
Supplement: Supplementary file 2 [file IPD-36-758-s002.docx]

Scopus
( TITLE-ABS-KEY ( "sodium fluoride" OR "sodium fluorides" OR "fluoride varnish" OR "fluoride varnishes" OR "fluoride varnishing" OR "fluoride application" OR "topical fluorides" ) ) AND ( TITLE-ABS-KEY ( "dental caries" OR "dental decay" OR "dental cavities" OR "cavities, dental" OR "cavity, dental" OR "decay, dental" OR "carious lesions" OR "carious lesion" OR "lesion, carious" OR "lesions, carious" OR "caries, dental" OR "dental cavity" OR "dental white spot" OR "spot, dental" OR "white spots, dental" OR "white dental" OR "white spots" OR "white spot, dental" OR "carious dentin" OR "carious dentins" OR "dentin, carious" OR "dentins, carious" ) ) AND ( TITLE-ABS-KEY ( pediatric OR paediatric OR child OR children OR preschool OR "pre school" OR kindergarten OR kindergarden OR "elementary school" OR "nursery school" OR "day care" OR schoolchild OR toddler OR boy OR boys OR girl OR girls OR "middle school" OR pubescent OR juvenile OR teen OR teenager OR youth OR "high school" OR adolescent OR "pre pubescent" OR prepubescent ) ) AND ( PUBYEAR > 2014 )

Web of Science

TS=("sodium fluoride" OR "sodium fluorides" OR "fluoride varnish" OR "fluoride varnishes" OR "fluoride varnishing" OR "fluoride application" OR "topical fluorides")

AND

TS=("dental caries" OR "dental decay" OR "dental cavities" OR "cavities, dental" OR "cavity, dental" OR "decay, dental" OR "carious lesions" OR "carious lesion" OR "lesion, carious" OR "lesions, carious" OR "caries, dental" OR "dental cavity" OR "dental white spot" OR "spot, dental" OR "white spots, dental" OR "white dental" OR "white spots" OR "white spot, dental" OR "carious dentin" OR "carious dentins" OR "dentin, carious" OR "dentins, carious")

AND

TS=(pediatric OR paediatric OR child OR children OR preschool OR "pre school" OR kindergarten OR kindergarten OR "elementary school" OR "nursery school" OR "day care" OR schoolchild OR toddler OR boy OR boys OR girl OR girls OR "middle school" OR pubescent OR juvenile OR teen OR teenager OR youth OR "high school" OR adolescent OR "pre pubescent" OR prepubescent)

AND

PY=2015-2025

Medline

| **#** | **Query** | **Results from 22 Jul 2025** |
| --- | --- | --- |
| 1 | fluorides, topical/ or sodium fluoride/ | 12,042 |
| 2 | ("sodium fluoride" or "sodium fluorides" or "fluoride varnish" or "fluoride varnishes" or "fluoride varnishing" or "fluoride application").ti,ab,kf. | 8,323 |
| 3 | exp child/ or exp infant/ or adolescent/ or exp pediatrics/ or child, abandoned/ or exp child, exceptional/ or child, orphaned/ or child, unwanted/ or minors/ or (pediatric* or paediatric* or (child* not childbearing) or baby or babies or preschool* or pre-school* or kindergarten* or kindergarden* or elementary school* or nursery school* or (day care* not adult*) or schoolchild* or toddler* or boy or boys or girl* or middle school* or pubescen* or juvenile* or teen* or youth* or high school* or adolesc* or pre-pubesc* or prepubesc*).mp. [mp=title, book title, abstract, original title, name of substance word, subject heading word, floating sub-heading word, keyword heading word, organism supplementary concept word, protocol supplementary concept word, rare disease supplementary concept word, unique identifier, synonyms, population supplementary concept word, anatomy supplementary concept word] | 4,972,993 |
| 4 | Dental Caries/ | 52,598 |
| 5 | ("dental caries" or "dental decay or dental cavities or cavities, dental or cavity, dental or decay, dental or carious lesions or carious lesion or lesion, carious or lesions, carious or caries, dental or dental cavity or dental white spot or spot, dental or white spots, dental or white dental or white spots or white spot, dental or white spots, dental or carious dentin or carious dentins or dentin, carious or dentins, carious").ti,ab,kf. | 27,816 |
| 6 | 1 or 2 | 15,769 |
| 7 | 4 or 5 | 60,750 |
| 8 | 3 and 6 and 7 | 2,321 |
| 9 | limit 8 to yr="2015 -Current" | 717 |

Embase

| **#** | **Query** | **Results from 22 Jul 2025** |
| --- | --- | --- |
| 1 | fluoride varnish/ | 4,215 |
| 2 | (fluorides, topical or "fluoride varnishes" or "fluoride varnishing" or "fluoride varnished").mp. [mp=title, abstract, heading word, drug trade name, original title, device manufacturer, drug manufacturer, device trade name, keyword heading word, floating subheading word, candidate term word] | 363 |
| 3 | 1 or 2 | 4,285 |
| 4 | exp child/ or exp infant/ or adolescent/ or exp pediatrics/ or child, abandoned/ or exp child, exceptional/ or child, orphaned/ or child, unwanted/ or minors/ or (pediatric* or paediatric* or (child* not childbearing) or baby or babies or preschool* or pre-school* or kindergarten* or kindergarden* or elementary school* or nursery school* or (day care* not adult*) or schoolchild* or toddler* or boy or boys or girl* or middle school* or pubescen* or juvenile* or teen* or youth* or high school* or adolesc* or pre-pubesc* or prepubesc*).mp. [mp=title, abstract, heading word, drug trade name, original title, device manufacturer, drug manufacturer, device trade name, keyword heading word, floating subheading word, candidate term word] | 5,428,833 |
| 5 | 3 and 4 | 2,025 |
| 6 | dental caries/ | 63,220 |
| 7 | ("caries" or caries, dental or "carious teeth" or "dental caries susceptibility" or "dental decay" or "tooth caries" or "tooth decay").mp. [mp=title, abstract, heading word, drug trade name, original title, device manufacturer, drug manufacturer, device trade name, keyword heading word, floating subheading word, candidate term word] | 76,508 |
| 8 | 6 or 7 | 76,508 |
| 9 | 5 and 8 | 1,682 |
| 10 | limit 9 to yr="2015 -Current" | 752 |

Central

| **#** | **Query** | **Results from 22 Jul 2025** |
| --- | --- | --- |
| 1 | fluorides, topical/ or sodium fluoride/ | 1,892 |
| 2 | ("sodium fluoride" or "sodium fluorides" or "fluoride varnish" or "fluoride varnishes" or "fluoride varnishing" or "fluoride application").mp. [mp=title, original title, abstract, floating sub-heading word, mesh headings, heading words, keyword] | 2,713 |
| 3 | ((exp child/ or exp infant/ or exp adolescent/ or pediatrics/ or "child, abandoned"/ or "child, exceptional"/ or "child, orphaned"/ or "child, unwanted"/ or minor/ or congenital, hereditary.mp.) and neonatal diseases.mp. and abnormalities/) or (pediatric* or paediatric* or (child* not childbearing) or preschool* or (pre adj school*) or kindergarten* or kindergarden* or (elementary adj school*) or (nursery adj school*) or ((day adj care*) not adult*) or schoolchild* or toddler* or boy or boys or girl* or (middle adj school*) or pubescen* or juvenile* or teen* or youth* or (high adj school*) or adolesc* or (pre adj pubesc*) or prepubesc*).ti,ab,kw. or (child* or adolesc* or pediat* or paediat*).jn. [mp=title, original title, abstract, floating sub-heading word, mesh headings, heading words, keyword] | 248,147 |
| 4 | Dental Caries/ | 3,737 |
| 5 | ("dental caries" or "dental decay or dental cavities or cavities, dental or cavity, dental or decay, dental or carious lesions or carious lesion or lesion, carious or lesions, carious or caries, dental or dental cavity or dental white spot or spot, dental or white spots, dental or white dental or white spots or white spot, dental or white spots, dental or carious dentin or carious dentins or dentin, carious or dentins, carious").ti,ab,kf. | 2,893 |
| 6 | 1 or 2 | 3,055 |
| 7 | 4 or 5 | 5,877 |
| 8 | 3 and 6 and 7 | 670 |
| 9 | limit 8 to yr="2015 -Current" | 350 |
